# SDN-1/Syndecan Acts in Parallel to the Transmembrane Molecule MIG-13 to Promote Anterior Neuroblast Migration

**DOI:** 10.1534/g3.115.018770

**Published:** 2015-05-28

**Authors:** Lakshmi Sundararajan, Megan L. Norris, Erik A. Lundquist

**Affiliations:** Programs in Genetics and Molecular, Cellular, and Developmental Biology, Department of Molecular Biosciences, University of Kansas, Lawrence, Kansas 66045

**Keywords:** MAB-5/Hox, MIG-13, Q neuroblasts, SDN-1/syndecan, neuronal migration

## Abstract

The Q neuroblasts in *Caenorhabditis elegans* display left-right asymmetry in their migration, with QR and descendants on the right migrating anteriorly, and QL and descendants on the left migrating posteriorly. Initial QR and QL migration is controlled by the transmembrane receptors UNC-40/DCC, PTP-3/LAR, and the Fat-like cadherin CDH-4. After initial migration, QL responds to an EGL-20/Wnt signal that drives continued posterior migration by activating MAB-5/Hox activity in QL but not QR. QR expresses the transmembrane protein MIG-13, which is repressed by MAB-5 in QL and which drives anterior migration of QR descendants. A screen for new Q descendant AQR and PQR migration mutations identified *mig-13* as well as *hse-5*, the gene encoding the glucuronyl C5-epimerase enzyme, which catalyzes epimerization of glucuronic acid to iduronic acid in the heparan sulfate side chains of heparan sulfate proteoglycans (HSPGs). Of five *C. elegans* HSPGs, we found that only SDN-1/Syndecan affected Q migrations. *sdn-1* mutants showed QR descendant AQR anterior migration defects, and weaker QL descendant PQR migration defects. *hse-5* affected initial Q migration, whereas *sdn-1* did not. *sdn-1* and *hse-5* acted redundantly in AQR and PQR migration, but not initial Q migration, suggesting the involvement of other HSPGs in Q migration. Cell-specific expression studies indicated that SDN-1 can act in QR to promote anterior migration. Genetic interactions between *sdn-1*, *mig-13*, and *mab-5* suggest that MIG-13 and SDN-1 act in parallel to promote anterior AQR migration and that SDN-1 also controls posterior migration. Together, our results indicate previously unappreciated complexity in the role of multiple signaling pathways and inherent left-right asymmetry in the control of Q neuroblast descendant migration.

Directed neuronal migration is an essential part of nervous system development. The Q neuroblasts, QR and QL, are an excellent system in which to study neuronal cell migration (Middelkoop and Korswagen 2014). The Q neuroblasts are born in the posterior lateral region of the worm (Sulston and Horvitz 1977; Chalfie and Sulston 1981). QR, on the right, extends anterior protrusions over the seam cell V4 whereas QL, on the left, protrudes posteriorly over the seam cell V5. The cell bodies then migrate atop the respective seam cells, and the Q cells undergo their first division (Honigberg and Kenyon 2000; Chapman *et al.* 2008). The daughter cells of QR and QL undergo further directional migration, divisions, and cell death to give rise to three neurons each. The QR descendants SDQR, AVM, and AQR migrate anteriorly, with AQR migrating the farthest to near the anterior deirid and first pharyngeal bulb (Chapman *et al.* 2008). The QL descendants SDQL, PVM, and PQR migrate posteriorly, with PQR migrating the farthest to behind the anus to the phasmid ganglion (Sulston and Horvitz 1977; White *et al.* 1986; Chapman *et al.* 2008).

Q migration occurs in two distinct phases. The directional migration of early QR and QL is directed by transmembrane proteins UNC-40/DCC, PTP-3/LAR, MIG-21, and CDH-4/Fat (Honigberg and Kenyon 2000; Middelkoop *et al.* 2012; Sundararajan and Lundquist 2012; Sundararajan *et al.* 2014). After this initial migration, QL and QR descendant migration is governed by EGL-20/Wnt signaling. QL descendants respond to an EGL-20/Wnt signal by activating expression of the *mab-5/Hox* gene, which drives further posterior migration (Kenyon 1986; Salser and Kenyon 1992; Chalfie 1993; Harris *et al.* 1996; Whangbo and Kenyon 1999; Korswagen *et al.* 2000; Herman 2001; Eisenmann 2005). QR descendants do not respond to the EGL-20/Wnt signal and do not activate *mab-5/Hox* and thus continue anterior migration.

Previous work has shown that EGL-20/Wnt and MAB-5/Hox are not required to direct early anteroposterior Q migrations (Chapman *et al.* 2008). However, MAB-5 is both necessary and sufficient to direct QL descendant migrations posteriorly (Kenyon 1986; Salser and Kenyon 1992). In *mab-5* loss-of-function, both AQR and PQR migrate anteriorly to the normal location of AQR. In a *mab-5* gain-of-function background, both QR and QL descendants migrate posteriorly to the normal position of PQR (Chapman *et al.* 2008; Tamayo *et al.* 2013).

The transmembrane molecule MIG-13 drives QR descendant anterior migration (Sym *et al.* 1999; Wang *et al.* 2013). MIG-13 expression is dependent on LIN-39/Hox, expression of which is inhibited by MAB-5/Hox (Sym *et al.* 1999; Wang *et al.* 2013). Thus, MAB-5 directs posterior migration by inhibiting *lin-39* and *mig-13*, genes involved in anterior migration. *mig-13* drives anterior Q descendant migrations in QR descendants that do not express *mab-5*, and in QL descendants in *mab-5* mutants (Sym *et al.* 1999; Wang *et al.* 2013). MIG-13 is a single-pass transmembrane protein a CUB (C1r/C1s, Uegf, Bmp1) domain and a low-density lipoprotein receptor repeat (Sym *et al.* 1999). *mig-13* acts cell-autonomously in QR descendant migration and might act as a receptor to polarize the actin cytoskeleton in response to guidance cues (Wang *et al.* 2013).

We conducted a genome-wide screen for new mutations affecting AQR and PQR migration. The screen identified *hse-5*, which encodes the *C. elegans* ortholog of the glucuronyl C5-epimerase enzyme, which catalyzes epimerization of glucuronic acid to iduronic acid in the heparan sulfate side chains of heparan sulfate proteoglycans (HSPGs) (Lindahl *et al.* 1998). Previous work has implicated HSPGs and modifying enzymes in cell migration and axon guidance (Merz *et al.* 2003; Rhiner *et al.* 2005; Hudson *et al.* 2006; Bulow *et al.* 2008; Schwabiuk *et al.* 2009; Wang *et al.* 2012; Gysi *et al.* 2013; Diaz-Balzac *et al.* 2014; Wang *et al.* 2015). HSPGs have long chains of differentially modified sugar side chains that play different roles in nervous system development (Minniti *et al.* 2004; Rhiner *et al.* 2005; Hudson *et al.* 2006; Johnson *et al.* 2006; Bulow *et al.* 2008; Wang *et al.* 2012). Indeed, HSE-5 is required for early Q neuroblast protrusion and migration along with the HSPG LON-2 (Wang *et al.* 2015).

The HSPG SDN-1/Syndecan is the only Syndecan in the *C. elegans* genome (Rhiner *et al.* 2005). SDN-1 is a transmembrane protein with heparan sulfate side chains attached to the extracellular domains and an intracellular PDZ binding domain (Lindahl *et al.* 1998; Bernfield *et al.* 1999; Esko and Selleck 2002). SDN-1 is necessary for the migration of different neuronal cell types such as the HSN, ALM, and CAN neurons and is expressed extensively in the nervous system (Rhiner *et al.* 2005). We found that *sdn-1* affected AQR, similar to *mig-13*. *sdn-1* also displayed weak PQR migration defects. This result combined with double mutant analysis with *mab-5* indicated that SDN-1 also regulates posterior migration. No defects in initial Q neuroblast migrations were observed in *sdn-1* mutants, whereas *hse-5* mutants displayed early migration defects, suggesting the involvement of other HSPGs in initial migration. Cell-specific expression revealed that SDN-1 can function autonomously in QR to promote AQR anterior migration. The other HSPGs LON-2, GPN-1/Glypican, and UNC-52/Perlecan had no detectable role in Q descendant migrations, nor did the *sdn-1gpn-1lon-2* triple mutant. We also identified an allele of *mig-13* that had AQR defects, as expected. Double mutant analysis revealed that MIG-13 and SDN-1 act in parallel in QR to promote AQR anterior migration.

## Materials and Methods

### *C. elegans* genetics

All experiments were conducted at 20° using standard *C. elegans* techniques (Sulston and Hodgkin 1988). The following strains and transgenes were used: LGI *cle-1(gk364)*; LGIII *hse-5(ok2463)*, *hse-5(lq49)*, *hse-5(tm472)*, *mab-5(e1239)*, *mab-5(gk670)*, *mab-5(e1751)*; LG IV *lqIs80[Pscm*::*gfp*::*caax]*; LGV *lqIs58[Pgcy-32*::*cfp]*; and LGX *sdn-1(zh20)*, *sdn-1(ok244)*, *mig-13(mu225)*, *mig-13(lq71)*, *lon-2(e678)*, *gpn-1(ok377)*. The presence of HSPG mutants were confirmed by polymerase chain reaction and/or sequencing. Transgenes were constructed using the standard gonadal microinjection techniques to produce extra-chromosomal arrays (Mello and Fire 1995).

### AQR/PQR mutant screen and mapping

L4 and young adult hermaphrodites of the strain LE2500, consisting of the *Pgcy-32*::*cfp* transgene *lqIs58* and the *Pscm*::*gfp* transgene *lqIs80*, were mutagenized with ethylmethane sulfonate using standard techniques (Anderson 1995). Mutagenized animals were placed on single seeded NGM plates and allowed to self-fertilize. F1 animals were placed on plates, three animals per plate. The F2 progeny were screened, using a fluorescence dissecting microscope, for animals with misplaced AQR and/or PQR visualized by the *lqIs58[Pgcy-32*::*cfp]* transgene. Defective animals were placed on single plates, and their progeny examined for AQR and PQR defects to ensure germline mutation. Approximately 3000 haploid genomes were screened.

New mutations were mapped using single nucleotide polymorphism mapping or next generation sequencing combined with snp mapping using the Cloudmap pipeline and the polymorphic CB4856 Hawaiian strain (Davis *et al.* 2005; Minevich *et al.* 2012).

### Plasmids

The cDNA yk139f3 encodes a full-length *sdn-1* cDNA. The *sdn-1* cDNA was positioned downstream of the *seam cell promoter (Pscm)* expressed in the hypodermal seam cells and Q cells (Chapman *et al.* 2008), and the Q cell−specific *egl-17* promoter (Branda and Stern 2000; Cordes *et al.* 2006; Sundararajan and Lundquist 2012; Sundararajan *et al.* 2014). Sequences of these plasmids are available upon request.

### Scoring AQR and PQR defects

Previously described quantification techniques were used (Chapman *et al.* 2008; Sundararajan and Lundquist 2012). The *gcy-32*:: *cfp* transgene expressed in the URX, AQR, and PQR neurons was used to visualize and score the final positions of AQR and PQR. The position of AQR and PQR are scored in five positions along the body of the worm (Table 1, Table 2, Table 3, Table 4, and Table 5). Position 1 is the wild-type position of AQR in the anterior deirid just posterior to the pharyngeal bulb. Position 2 represents the region posterior to the pharyngeal bulb and anterior to the vulva. Position 3 represents the region around the vulva. Position 4 represents the region posterior to the vulva near the posterior deirid. The Q cells are born and undergo their initial polarizations and migrations in this position. Position 5 represents the region behind the anus in the phasmid ganglion, which is the wild-type position of PQR. AQR and PQR were scored and analyzed in L4 larval animals, which is after AQR and PQR undergo their final migrations. At least 100 animals were scored for each genotype and statistical significance was determined using Fisher’s exact test. The predicted additive phenotype of double mutants for comparison in Fisher’s exact test was calculated by the formula p(A) = p1 + p2 – (p1p2), where p(A) is the predicted additive proportion, p1 is the proportion in single mutant 1, and p2 is the proportion in single mutant 2.

**Table 1:** HSE-5 and MIG-13 control AQR and PQR migration

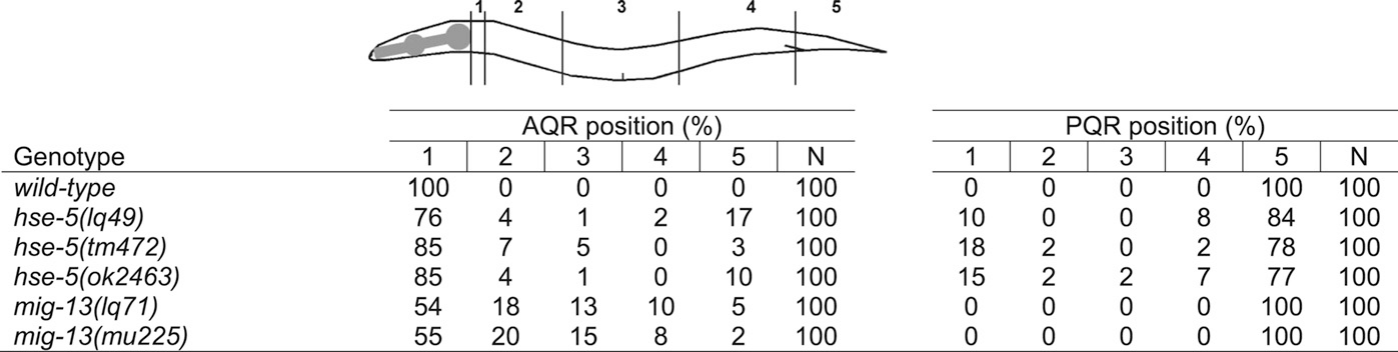

**Table 2:** SDN-1/syndecan controls AQR and PQR migration

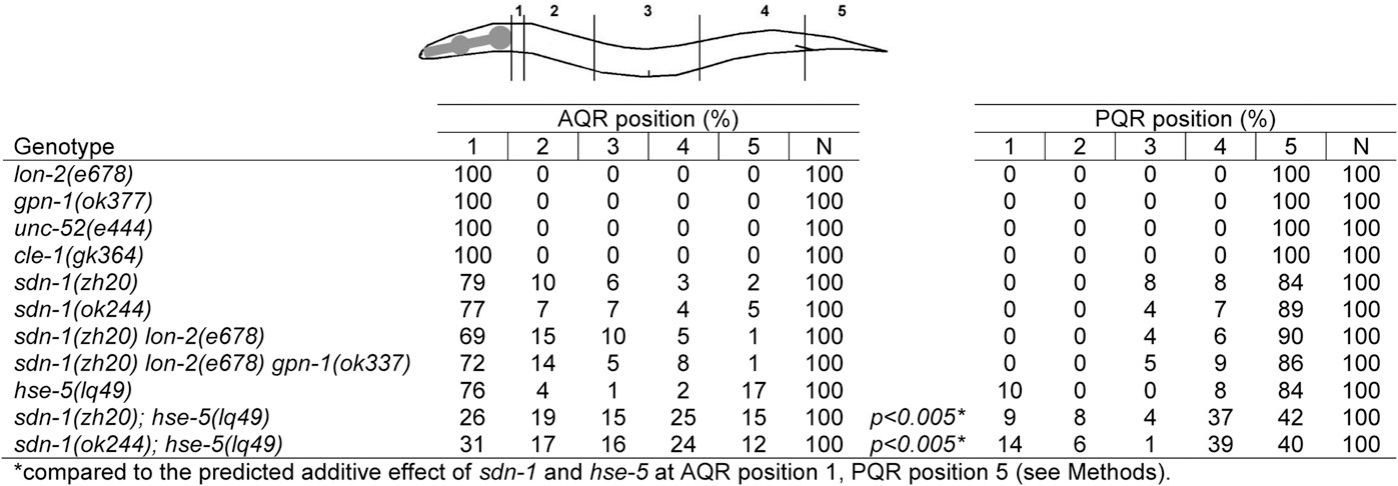

**Table 3:** SDN-1 can act in the QR for anterior migration

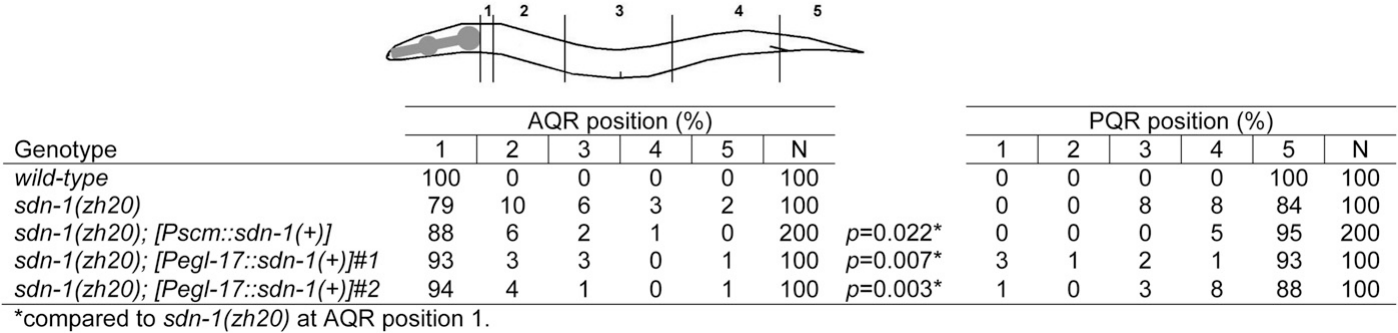

**Table 4:** SDN-1 and MIG-13 act redundantly in AQR migration

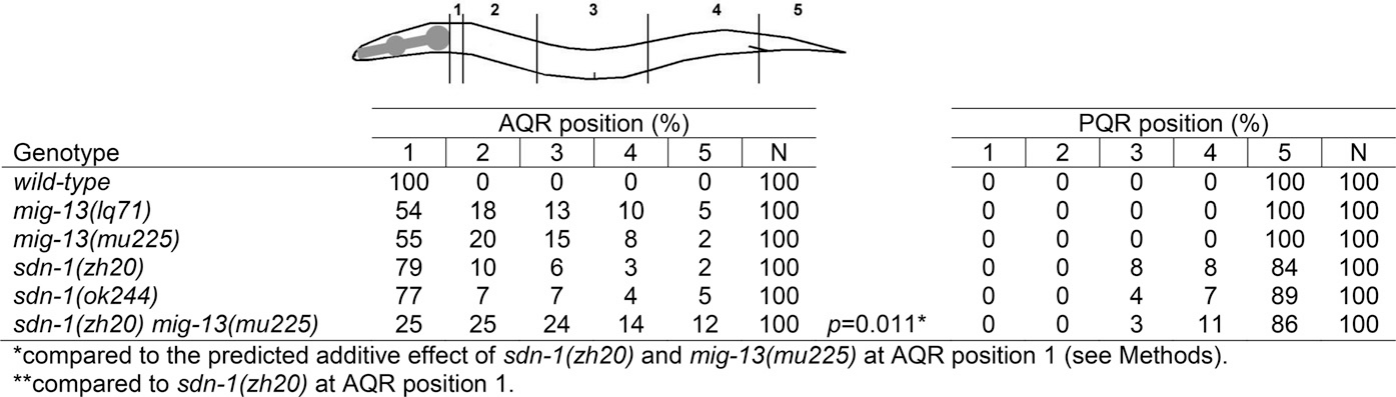

**Table 5:** MAB-5 interacts genetically with SDN-1 and MIG-13

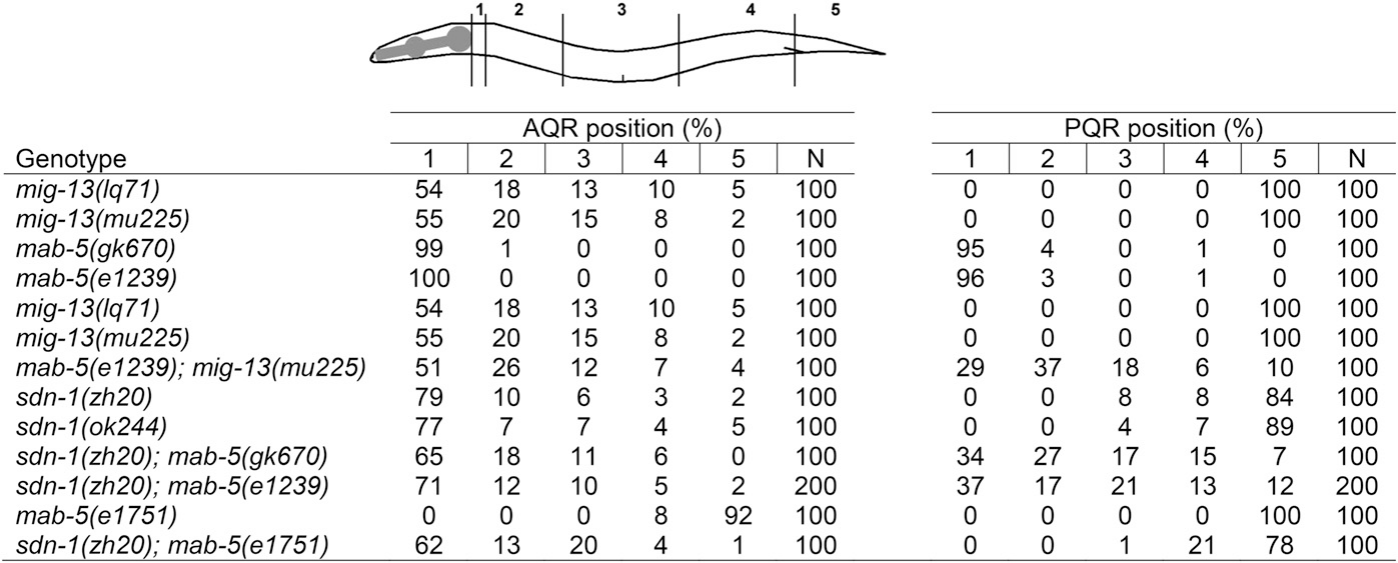

### Scoring early QR and QL defects

**Figure 1:**
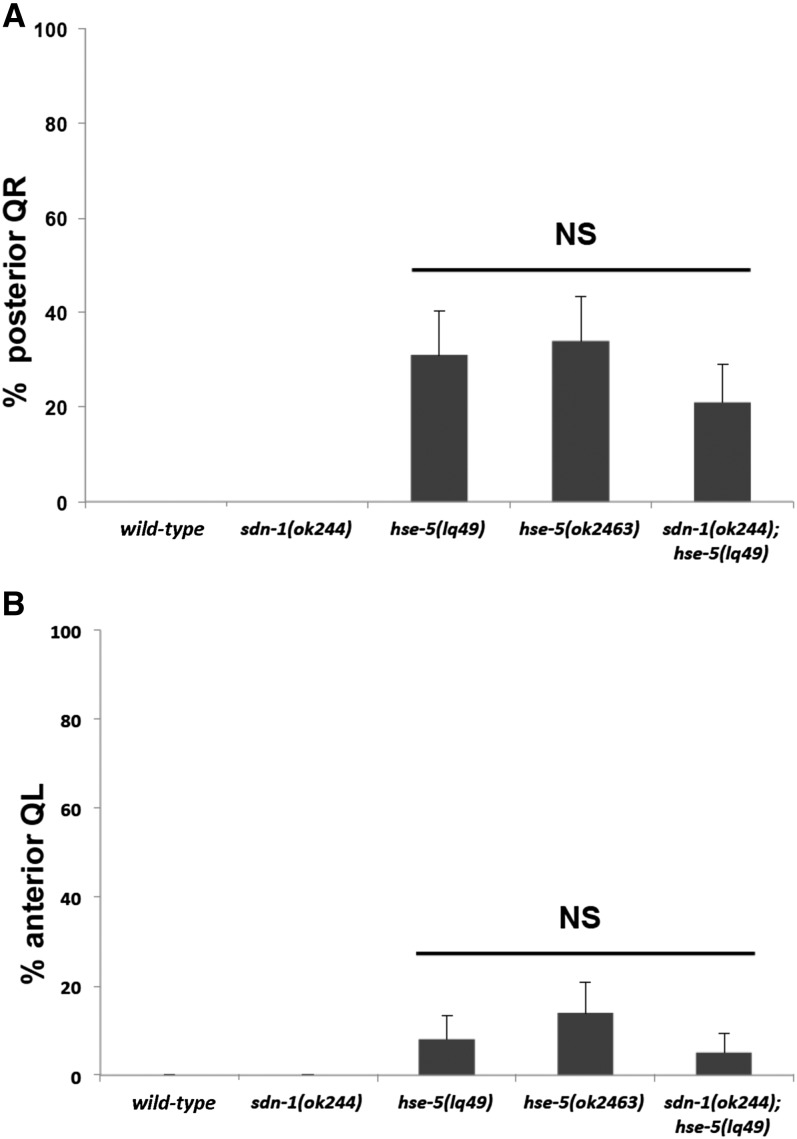
Quantification of early QR and QL defects in *sdn-1* and *hse-5* single and double mutants: Graphs represent the division stage of QR and QL at 4−4.5 hr posthatching. Genotype is on the X-axis, and the percentage of defective posterior QR and anterior QL migration and division is the Y-axis. Migration was scored as defective when QR divided posterior on the seam cell V5 and when QL divided anterior on the seam cell V4. The error bars represent two times the standard error of proportion, and the statistical difference between the genotypes were determined by Fisher’s exact test. Twenty-five animals or more were scored for each genotype. (A) QR defects in *sdn-1*, *hse-5*, and the double mutants. (B) QL defects in *sdn-1*, *hse-5*, and the double mutants.

Previously described larval synchronization techniques were used (Chapman *et al.* 2008; Dyer *et al.* 2010; Sundararajan and Lundquist 2012). All adult and larval worms were washed from plates using M9 buffer when the eggs remain adhered to the plates. These eggs were allowed to hatch and the larvae were collected every half an hour. All the larvae were collected between 0 and 0.5 hr after hatching. The larvae were then staged and visualized at 2−2.5, 3−3.5, and 4−4.5 hr posthatching using the *Pscm*::*gfp*::*caax* transgene. The wild-type larvae were visualized at 2-2.5 hr posthatching, and they exhibited defined anterior QR protrusions and posterior QL protrusions. These protrusions extended over the seam cells V4 and V5, respectively. At approximately 3−3.5 hr posthatching, QR and QL follow the protrusions and migrate on top of the seam cells. QR migrates anterior on the seam cell V4 and QL migrates posterior on the seam cell V5. The larvae visualized at 4−4.5 hr posthatching showed QR and QL undergoing their first round of division. QR divides on V4 and QL divides on V5. Defects in direction of protrusion, migration, and division stages were scored for all the genotypes. QR that protruded, migrated, and divided posterior on the seam cell V5 were scored as defective. QL that protruded, migrated, and divided anterior on the seam cell V4 were scored as defective. Defects observed in QR and QL at 4−4.5 hr post hatching are represented in Figure 1. Previous work has shown that the defects seen in protrusion and migration stages do not differ significantly from the division stage. At least 25 cells were scored for each genotype and statistical significance was determined with the Fisher’s exact test.

## Results

### A screen for new mutations affecting AQR and PQR migration identified *hse-5* and *mig-13*

In a genome-wide screen for mutations affecting AQR and PQR migration (see the section *Materials and Methods*), we identified *hse-5(lq49)* and *mig-13(lq71)* (Table 1). *hse-5(lq49)* caused directional migration defects in both AQR and PQR. The *hse-5(lq49)* lesion was mapped and identified by whole-genome resequencing and the Cloudmap protocol (Minevich *et al.* 2012). *lq49* was linked to linkage group III and resulted in a glutamine 172 to stop (C to T) in the *hse-5* gene. The previously-isolated *hse-5(ok2463)* and *hse-5(tm472)* mutations also showed AQR and PQR directional defects similar to *hse-5(lq49)* (Table 1). *hse-5* encodes the *C. elegans* ortholog of the glucuronyl C5-epimerase enzyme, which catalyzes epimerization of glucuronic acid to iduronic acid in the heparan sulfate side chains of HSPGs. *hse-5* was shown previously to affect axon guidance and cell migration (Lindahl *et al.* 1998; Rhiner *et al.* 2005; Bulow *et al.* 2008), including Q neuroblast migration (Wang *et al.* 2015). Our results indicate that *hse-5* controls the direction and extent of AQR and PQR migration.

*mig-13(lq71)* was mapped to the X linkage group by the use of single-nucleotide polymorphism mapping (Davis *et al.* 2005). *mig-13(lq71)* affected only AQR and not PQR migration (Table 1), similar to the known effects of the X-linked *mig-13* on the QR but not QL lineage (Sym *et al.* 1999; Wang *et al.* 2013). As expected, the existing *mig-13(mu225)* mutation showed AQR-specific defects (Table 1), and *mig-13(lq71)* failed to complement *mig-13(mu225)* for AQR defects (data not shown). *mig-13* encodes a transmembrane protein with an extracellular C1r/C1s, Uegf, Bmp1 (CUB) domain and a low-density lipoprotein receptor repeat and a proline-rich cytoplasmic tail (Sym *et al.* 1999). These results indicate that HSPGs and the transmembrane protein MIG-13 regulate Q descendant migration.

### SDN-1/syndecan controls AQR anterior migration

HSPGs have a well-characterized role in axon pathfinding and cell migrations (Minniti *et al.* 2004; Rhiner *et al.* 2005; Hudson *et al.* 2006; Schwabiuk *et al.* 2009). Because *hse-5* caused AQR and PQR migration defects, we wanted to analyze the effect of other *C. elegans* HSPGs in AQR and PQR migrations. Mutations in *glp-2/Glypican*, *lon-2*, *cle-1/CollagenXVIII*, and *unc-52/Perlecan* did not affect AQR or PQR migration (Table 2). However, mutations in *sdn-1/Syndecan* affected the extent and direction of AQR migration and had a weak effect on the extent of PQR migration. The two well-characterized mutant alleles of *sdn-1*, *zh20* and *ok244* (Rhiner *et al.* 2005), are deletions that affect exons 1−5 and exons 2 and 3, respectively. *zh20* is a candidate for a null mutant, and *ok244* is thought to function as a hypomorph (Rhiner *et al.* 2005), but we observed similar defects in both. In *sdn-1* mutants, AQR failed to migrate all the way to its anterior wild-type position (23–24%) and in fact migrated posteriorly in 3–5% of animals. PQR migrations defects were weaker (11–16%), with defective cells failing to migrate away from their birthplace and possibly slightly to the anterior, but never to the normal anterior position of AQR as observed in *hse-5* (Table 2).

### *sdn-1* expression in Q cells is sufficient for anterior migration

We drove expression of the *sdn-1* cDNA under the control of the *Pscm* expressed in the hypodermal seam cells and Q cells (Chapman *et al.* 2008), and the Q cell-specific *egl-17* promoter (Branda and Stern 2000; Cordes *et al.* 2006; Sundararajan and Lundquist 2012; Sundararajan *et al.* 2014). Both *Pscm*::*sdn-1* and *Pegl-17*::*sdn-1* significantly rescued the AQR defects of *sdn-1(zh20)* (Table 3), indicating that *sdn-1* function in QR descendants is sufficient for anterior migration. Neither transgene rescued PQR migration defects observed in *sdn-1(zh20)*. In some *sdn-1(zh20)*; *Pegl-17*::*sdn-1* animals, PQR migrated anteriorly to the normal position of AQR, a defect not observed in *sdn-1* alone. Although *sdn-1* can act in QR for anterior descendant migration, its role in QL is more complex. *sdn-1* might act nonautonomously in QL descendant migration, or transgenic expression in QL might drive anterior migration.

### HSE-5 and SDN-1 act redundantly in AQR and PQR migration

Previous work has shown that in axon pathfinding and cell migration, HSE-5 functions with other HSPGs redundantly with SDN-1 (Rhiner *et al.* 2005). *hse-5* and *sdn-1* alone had comparable effects on AQR migration (Table 2). In *hse-5*; *sdn-1* double mutants, we observed a significant increase in the percentage of AQR migration defects compared with the predicted additive effects of the double mutant (Table 2). PQR migration defects also were significantly enhanced (Table 2). These data suggest that *hse-5* and *sdn-1* act redundantly to control AQR and PQR migration.

HSPGs and modifying enzymes have been shown to act additively and redundantly in some migration events (Rhiner *et al.* 2005; Diaz-Balzac *et al.* 2014; Kinnunen 2014). To investigate redundancy in AQR and PQR migration, we analyzed compound mutants of the genes encoding the other transmembrane HSPGs in the *C. elegans* genome, *lon-2* and *gpn-1*. Neither the *sdn-1(zh20) lon-2(e678)* double nor the *sdn-1(zh20) lon-2(e678) gpn-1(ok377)* triple mutant enhanced AQR and PQR defects of *sdn-1(zh20)* (Table 2).

### HSE-5, but not SDN-1, affects early Q protrusion and migration

QR and QL undergo an initial anterior and posterior protrusion and migration controlled by the transmembrane proteins UNC-40/DCC, PTP-3/LAR, and CDH-4/Fat-like cadherin (Honigberg and Kenyon 2000; Sundararajan and Lundquist 2012; Middelkoop and Korswagen 2014; Sundararajan *et al.* 2014). *hse-5(lq49)* disrupted early QR and QL migrations (Figure 1), as was observed previously for other *hse-5* mutations (Wang *et al.* 2015). In *hse-5(lq49)* mutants, QR migrated posteriorly and QL migrated anteriorly in some animals (Figure 1). *sdn-1* alone had no effect on QL and QR early protrusion and migration, nor did it modify the effects of *hse-5(lq49)* in double mutants (Figure 1). These results indicate that *hse-5* is required for early QL and QR migrations but *sdn-1* is not. HSE-5 is likely acting via other HSPGs in regulating early Q migration as well as redundantly with SDN-1 in Q descendant migration. The HSPG LON-2 might be a target of HSE-5 in early Q protrusion and migration (Wang *et al.* 2015), but did not act redundantly with *sdn-1* in AQR and PQR migration (Table 2).

HSE-5 acts outside of the Q cells to direct early Q migrations (Wang *et al.* 2015), and we found that SDN-1 can act in the QR to promote anterior AQR migration (Table 3). Possibly, SDN-1 acts redundantly with another HSPG modified by HSE-5 outside of the Q cells.

### SDN-1 and MIG-13 function redundantly in AQR migration

MIG-13 controls anterior migration of QR descendants (Sym *et al.* 1999; Wang *et al.* 2013). MIG-13 can act in the Q cells themselves to direct anterior migration (Wang *et al.* 2013). MIG-13 expression is inhibited in QL by the Hox transcription factor MAB-5 (Wang *et al.* 2013). QR descendants do not respond to the EGL-20/Wnt signal and thus do not activate MAB-5, resulting in MIG-13 expression in QR descendants (Wang *et al.* 2013). MIG-13 controls the anterior polarization and migration of the QR descendants (Wang *et al.* 2013). QL descendants respond to the EGL-20/Wnt signal by expressing MAB-5 and inhibiting MIG-13 expression, resulting in posterior migration.

*mig-13* and *sdn-1* both affected anterior AQR migration, with *mig-13* having a more severe effect (Table 4). The double-mutant *sdn-1*; *mig-13* had significantly increased AQR migration defects compared to the predicted additive effect of the mutations (Table 3), indicating a synergistic interaction and redundancy between *sdn-1* and *mig-13* in AQR migration. *mig-13* did not enhance PQR migration defects of *sdn-1*, indicating that MIG-13 redundancy with SDN-1 is limited to AQR. MIG-13 and SDN-1 act in parallel to promote anterior migration in AQR.

### *mig-13* and *sdn-1* interact genetically with *mab-5/Hox*

Previous work has shown that MAB-5/Hox inhibits MIG-13 expression in QL descendants (Wang *et al.* 2013). MAB-5/Hox expression is cell-autonomously required to direct posterior QL descendant migration, including PQR (Kenyon 1986; Chapman *et al.* 2008; Tamayo *et al.* 2013). In *mab-5(e1239)* and *mab-5(gk670)* loss-of-function backgrounds, both AQR and PQR migrated anteriorly to the wild-type AQR position (Table 5). In a *mig-13(mu225)*; *mab-5(e1239)* double mutant, AQR defects resembled *mig-13* alone. *mig-13* significantly suppressed anterior migration of PQR compared with a *mab-5* loss of function background alone (Table 5), with some PQR (10%) migrating posteriorly. These results indicate that anterior migration of PQR in *mab-5* mutants is partially dependent upon *mig-13*. This result is consistent with previous observations that MIG-13 is ectopically expressed in QL descendants in *mab-5* mutants through LIN-39/Hox activity (Wang *et al.* 2013). *sdn-1(zh20)* double mutants with *mab-5* had a similar effect (Table 4) in that AQR and PQR often failed in their anterior migrations in the double mutants, indicating that SDN-1 is also required for anterior PQR migration in *mab-5* mutants.

In a *mab-5(e1751)* gain-of-function background, AQR migrated posteriorly as previously reported (Table 5). Surprisingly, *sdn-1* suppressed posterior AQR migration of the *mab-5(e1751)* gain-of-function mutant, indicating that *sdn-1* is required for posterior migration of AQR in *mab-5(e1751)* gain-of-function. These data indicate that SDN-1 is required for both anterior and posterior migration, consistent with weak PQR defects observed in *sdn-1* mutants.

## Discussion

### SDN-1/syndecan regulates Q descendant migrations

The guidance of Q descendants in the anteroposterior axis involves intrinsic left-right asymmetries of the QL and QR cells, as well as extracellular signals, such as Wnts (Zinovyeva *et al.* 2008). HSPGs have been implicated considerably in nervous system development (Bulow *et al.* 2002; Minniti *et al.* 2004; Rhiner *et al.* 2005; Hudson *et al.* 2006; Johnson *et al.* 2006; Schwabiuk *et al.* 2009; Diaz-Balzac *et al.* 2014; Kinnunen 2014). The two classes of HSPGs, the syndecans and glypicans, are known to regulate cell migration and axon pathfinding in the nervous system (Rhiner *et al.* 2005; Hudson *et al.* 2006; Bulow *et al.* 2008). In *C. elegans*, SDN-1 is the only Syndecan encoded by the genome (Rhiner *et al.* 2005). Previous work has shown that SDN-1 is required to direct neuronal cell migration, including HSN, CAN, and ALM neurons (Rhiner *et al.* 2005; Kinnunen 2014). It also is required for proper axon pathfinding (Rhiner *et al.* 2005). HSPGs have heparan side chains that require modifications in a function-dependent manner (Lindahl *et al.* 1998). HSE-5 inhibits N-sulfation and stimulates 2-O and 6-O-sulfation (Townley and Bulow 2011). SDN-1 interacts with HS-modifying genes including *hse-5* in a context-dependent manner, acting in the same pathway or in parallel pathways (Lindahl *et al.* 1998; Bulow and Hobert 2004; Rhiner *et al.* 2005; Bulow *et al.* 2008; Kinnunen 2014).

Here we show that SDN-1/syndecan is required for proper extent and direction of migration of QR descendant AQR, with weaker effects on the migration of PQR. *sdn-1* is expressed broadly in neuronal and non-neuronal cells, including hypodermis and pharynx (Minniti *et al.* 2004; Rhiner *et al.* 2005; Hudson *et al.* 2006). We show that SDN-1 can function in QR to promote anterior migration, suggesting a cell-autonomous function.

**Figure 2:**
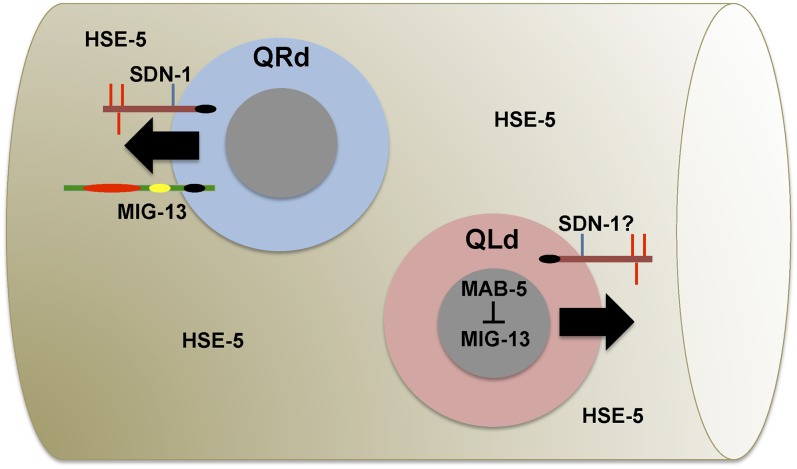
SDN-1 acts in parallel to MIG-13 in anterior AQR migration. Anterior is to the left, and dorsal is up. QRd represents QR descendants (*e.g.*, AQR), and QLd represents QL descendants (*e.g.*, PQR). Data presented here suggest that MIG-13 and SDN-1 act in parallel in QR to promote anterior migration. In QL, MAB-5 inhibits MIG-13 expression, resulting in posterior migration (Wang *et al.* 2013). The role of SDN-1 in QL migration is unclear. It could act outside of QL, or transgenic expression might perturb its function in QL. HSE-5 might modify another HSPG that acts redundantly with SDN-1, possibly outside of the Q cells. On the SDN-1 depiction, the black circle represents the PDZ-binding region, the red lines heparan sulfate chains, and the green lines chondroitin sulfate chains. On the MIG-13 depiction, the red circle represents the C1r/C1s, Uegf, Bmp1 (CUB) domain, the yellow the low-density lipoprotein repeat, and the black the proline-rich region.

*hse-5* mutants also displayed both AQR and PQR migration defects, and *hse-5*; *sdn-1* double mutants showed synergistically more severe defects in both AQR and PQR migration, suggesting redundant function. *hse-5* is expressed in hypodermis (Bulow and Hobert 2004), is not expressed in Q cells, and is required in the hypodermis for early Q cell migration (Wang *et al.* 2015). Thus, *hse-5* acts nonautonomously in Q migration. HSE-5 might modify another HSPG that acts redundantly with SDN-1. No other single mutations in the *lon-2*, *gpn-1*, *unc-52*, or *cle-1* genes encoding HSPGs showed any effect on AQR and PQR. The HSPGs LON-2 and GPN-1/Glypican are both, like SDN-1, transmembrane molecules, and the HSPGs CLE-1, UNC-52, and AGR-1/Agrin are predicted secreted molecules. The “transmembrane” triple mutant *sdn-1lon-2gpn-1* showed no more AQR defects than *sdn-1* alone, arguing against redundant function of LON-2 and GPN-1 with SDN-1 in AQR migration. Possibly, HSE-5 modifies one or more of the HSPGs outside of the Q cells that interact redundantly with SDN-1 in AQR and PQR migration (Figure 2). This is distinct from the roles of *sdn-1*, *lon-2*, and *gpn-1* in another cell migration event, that of the HSN neuron, in which each additively controls HSN migration (Kinnunen 2014). This indicates that different migration events use distinct mechanisms involving HSPGs to control migration.

*hse-5* mutants also had defects in initial QL and QR protrusion and migration as described previously (Wang *et al.* 2015). *sdn-1* mutations alone had no initial QL or QR migration defects, nor did *sdn-1* enhance the initial QR and QL defects of *hse-5* mutants. One interpretation of this result is that HSE-5 and SDN-1 act in the same pathway in early protrusion, and that HSE-5 also regulates another HSPG in parallel to SDN-1 in initial migration. Indeed, genetic interactions suggest that the HSPG LON-2 might be a target of HSE-5 in initial Q migrations: *lon-2* mutants alone showed no phenotype but suppressed *hse-5* (Wang *et al.* 2015). Alternately, SDN-1 might not affect early Q migrations, only later Q descendant migrations.

### SDN-1/syndecan acts in parallel to MIG-13 in AQR to direct anterior migration

*sdn-1* and *mig-13* single mutants had similar AQR migration defects. *sdn-1mig-13* double mutants showed synergistically increased AQR migration defects (Table 4). This finding suggests that SDN-1 and MIG-13 act redundantly in anterior AQR migration. Previous work has shown that MIG-13 is required autonomously in QR descendants to direct their anterior migration (Wang *et al.* 2013) and is repressed by MAB-5 in QL to allow posterior migration. MIG-13 acts in QR descendants, possibly as a receptor, to drive anterior polarization and migration (Wang *et al.* 2013). SDN-1 might also act in parallel to MIG-13 to drive anterior migration (Figure 2). One model is that SDN-1 might be generally required for migration in both anterior and posterior directions, whereas MIG-13 cooperates with SDN-1 in anterior, but not posterior migration. This is consistent with weak PQR defects in *sdn-1* mutants. However, transgenic expression of *sdn-1* in QL did not rescue PQR defects, suggesting that SDN-1 might function nonautonomously in QL descendant migration. *Pegl-17*::*sdn-1(+)* expression caused a small percentage of complete PQR anterior migration not seen in *sdn-1* alone (Table 2), suggesting that SDN-1 transgenic expression in QL might drive anterior migration. Although the role of SDN-1 in QL is unclear, our results show that SDN-1 can function in QR, in parallel to MIG-13, to promote anterior migration of the QR descendant AQR.

### SDN-1 mediates responses to MAB-5/Hox

In *mab-5* loss-of-function mutants, QL descendants migrate anteriorly, similar to QR descendants. We found that *sdn-1*; *mab-5* double mutants had defects in anteriorly migrating PQRs, similar to AQRs in an *sdn-1* single mutant. MIG-13 also was required for anterior migration of PQR in *mab-5* loss-of-function. Previous work showed that MAB-5 represses LIN-39 expression in QL, and thus MIG-13 also is not expressed in QL, so the QL descendants do not migrate anteriorly (Wang *et al.* 2013). Our data are consistent with this model and implicate SDN-1 in acting in parallel to MIG-13 to promote anterior migration.

*mab-5(e1751)* gain-of-function results in posterior migration of both QR and QL descendants, including AQR and PQR. *sdn-1(zh20)*; *mab-5(e1751)* showed much anterior AQR and PQR migration, suggesting that *sdn-1* is required for posterior migration in the *mab-5(e1751)* gain-of-function mutant, which might represent sensitized backgrounds that reveal the effect of SDN-1 on posterior migration. In sum, these data indicate that SDN-1/syndecan can regulate both anterior and posterior Q descendant migration controlled by MAB-5/Hox.

Our results reveal the complexity of the decision of the Q descendants to migrate anteriorly or posteriorly. Wnts have been shown to redundantly control Q descendant migrations (Zinovyeva *et al.* 2008). Here we show that the HSPG Syndecan is also involved, possibly acting in QR along with the transmembrane molecule MIG-13 to promote anterior migration (Figure 2). The role of SDN-1 in QL is unclear, but it might act outside of QL for posterior migration. Alternately, transgenic expression of *sdn-1* might perturb its function in QL. In dorsal migration of the gonadal distal tip cells, *sdn-1* mutation results in misregulated EGL-20/Wnt signaling (Schwabiuk *et al.* 2009). SDN-1 also interacts with Wnt signaling to orient spindle positioning in the early embryo (Dejima *et al.* 2014). It is possible that SDN-1 governs AQR and PQR migration by interacting with the Wnt signals that redundantly guide their anterior-posterior migrations (Zinovyeva *et al.* 2008). We show that SDN-1 can function in QR autonomously, so it is possible that SDN-1 modifies the manner in which QR descendants respond to Wnt signals. It is becoming clear from our work and that of others that anteroposterior Q descendant migrations are due to the intersection of multiple signaling pathways and the inherent left-right asymmetry of the cells, which mandates how the cells respond to these pathways.
